# Complete genome sequence of *Prevotella spp*. GTC17253 isolated from equine in Japan

**DOI:** 10.1128/mra.00854-24

**Published:** 2024-12-31

**Authors:** Masahiro Hayashi, Jun Yonetamari, Yoshinori Muto, Hidekazu Niwa, Kaori Tanaka

**Affiliations:** 1 Institute for Glyco-core Research iGCORE, Gifu University, Gifu, Japan; 2 Division of Anaerobe Research, Life Science Research Center, Gifu University, Gifu, Japan; 3 Gifu University Center for Conservation of Microbial Genetic Resource, Gifu, Japan; 4 Division of Clinical Laboratory, Gifu University Hospital, Gifu, Japan; 5 United Graduate School of Drug Discovery and Medical Information Sciences, Gifu University, Gifu, Japan; 6 Epizootic Research Center, Equine Research Institute, Japan Racing Association, Shimotsuke, Tochigi, Japan; Wellesley College Department of Biological Sciences, Wellesley, Massachusetts, USA

**Keywords:** *Prevotella*, equine

## Abstract

*Prevotella* is one of the major clinical anaerobic bacteria belonging to the family *Prevotellaceae*. Herein, we report the complete genome sequence of a clinical isolate of new *Prevotella* species obtained from equine bronchial lavage fluid in Japan. The genome comprised a circular chromosome with a length of 2,948,037 bp.

## ANNOUNCEMENT

Identifying closely related pet animal pathogens is important for understanding pathogenic factors through genome analysis (1, 2). The strain (GTC17253) was isolated from an equine with pneumonia in Hakodate, Hokkaido, Japan, in 2010. Bronchoalveolar lavage fluid was cultured on *Brucella* HK agar with 5% laked sheep blood at 37°C for 48 h under anaerobic conditions (82% N_2_, 10% CO_2_, and 8% H_2_) and submitted to our laboratory as a suspected *Prevotella* strain for further identification. API 32A (bioMérieux Japan Ltd., Tokyo, Japan) analysis confirmed its identification at the genus level (*Prevotella spp.*). The strain was stored at −80°C after several passages during identification.

The strain was cultured under the same conditions used for isolation. After incubation, the colonies were scraped, suspended in TE buffer, and pelleted through centrifugation. Genomic DNA was extracted from the pellet using NucleoBond® HMW DNA (MACHEREY-NAGEL, Japan).

To further identify the strain, the 16s rRNA gene was sequenced after amplification with PCR using primers 16S-8F (AGAGTTTGATCMTGGCTCAG) and 16S-1492R (GGYTACCTTGTTACGACTT). BLASTN analysis (3) of the PCR amplified full-length 16S rRNA gene identified the strain as *Prevotella spp*., most closely related to *Prevotella phocaeensis* DSM 103364T (Acc.no.LN998069) with 91.6% similarity.

The genome was sequenced using the PromethION 24 system (Oxford Nanopore Technologies, [ONT], Oxford, UK) for long-read sequencing and the NovaSeq 6000 system (Illumina Inc., San Diego, CA, USA) for short-read sequencing as previously described (4–6). The same DNA extract was used for both sequencing processes. A library was constructed using a ligation sequencing kit (SQK-LSK114; ONT) without shearing for long-read sequencing. Small DNA fragments were removed using Short Read Eliminator XS (Pacific Bioscience of California Inc., USA). Sequencing was performed using a PromethION 24 (ONT) on a FLO-PRO114M flow cell. Long-read sequence data were base-called with Guppy v.7.1.4 (super accuracy mode). The raw reads were trimmed and quality filtered using NanoFilt v.2.7.1 (7) with the parameters “-l 1000 -q 10—headcrop 50.” For short-read sequencing, the DNA Prep (M) Tagmentation kit (Illumina Inc.) was used for library construction. Subsequently, 2 × 151 bp paired-end sequencing was performed using the NovaSeq 6000 platform (Illumina Inc.). Raw sequencing reads were processed using fastp v.0.20.1 (8) with the parameters “-q 30 -n 20 -t 1 -T 1.” The short reads’ quality was assessed using fastp v.0.20.1 (8). The mean read quality of the long reads was scored using NanoPlot 1.32.1 (8). Short (over 94.2% of bases > Q30 averaged) and long (mean read quality of 14.0) reads were assembled using Unicycler v.0.4.8 (9) with default settings. Assembly circularization and rotation were performed automatically using the Unicycler. The assembly was rotated to start with the *dnaA* gene on the forward strand. The contig graph was visualized using Bandage v.0.8.1 (10). The integrity of the assembled genomic data was confirmed using blobtools v.1.0 (11).

Table 1 summarizes the genome information. The results of Unicycler and Bandage indicate that a complete circular genome was assembled. Quality assessment and genome statistics were computed using QUAST (v5.2.0) (12) and CheckM (v1.2.2) (13). CheckM indicated that the genome was 100% complete with 0.52% contamination. The DDBJ Fast Annotation and Submission Tool (14) was used to predict 2,369 coding sequences, 12 ribosomal RNAs, 56 transfer RNAs, and one CRISPR sequence. Species identification with an average nucleotide identity value of the genome sequence using GTDB-tK (v.2.3.2) was unsuccessful (15).

**TABLE 1:** Information regarding the complete genome sequence of a *Prevotella spp*. strain isolated from equine bronchial lavage fluid in Japan

	Strain name
Parameter	*Prevotella spp*. GTC17253
NovaSeq6000 sequencing^*a*^	
No. of reads	3,190,318
Size (kb)	467,029
Avg coverage (x)	158
DRA accession no.	DRR584460
ONT sequencing^*a*^	
No. of reads	136,891
Size (kb)	1,231,213
Avg read length (bp)	8,994
Avg coverage (x)	418
N50	20,295
DRA accession no.	DRR584465
Assembly	
Assembly N50 (bp)^*c*^	2,948,037
Estimated genome completeness (%)^*d*^	100.0
Estimated genome contamination (%)^*d*^	0.52
Genome structure	One chromosome
DDBJ/GenBank accession no.	AP035785
Genome size (bp)	2,948,037
GC content (%)	
(chromosome/ plasmid name)	46.7
No. of coding sequences^*b*^	2,369
Number of rRNAs^*b*^	12
Number of tRNAs^*b*^	56
Number of CRISPRs^*b*^	1

^*a*^ DRA, DDBJ Sequence Read Archive.

^*b*^ DFAST, DDBJ Fast Annotation and Submission Tool.

^*c*^ Determined with Quast.

^*d*^ Determined with CheckM.

## Data Availability

The genome sequences of *Prevotella spp*. (GTC17253) strains from Japanese equine have been deposited in DDBJ (https://www.ddbj.nig.ac.jp/index.html) with accession number AP035785. Raw sequence data for GTC17253 have been deposited in the DDBJ/Sequence Read Archive with accession numbers DRR584460 and DRR584465.
